# Bilateral Proximal Tibiofibular Synostosis Caused by Osteochondroma in a 21-Year-Old Highly Active Male—First in Literature

**DOI:** 10.3390/medicina57101126

**Published:** 2021-10-18

**Authors:** Lorenz Pisecky, Gerhard Großbötzl, Stella Stevoska, Christian Stadler, Maximilian Ziernhöld, Petar Noack, Tobias Gotterbarm, Matthias Luger

**Affiliations:** Department for Orthopedics and Traumatology, Kepler University Hospital GmbH, Johannes Kepler University Linz, Krankenhausstraße 9, 4020 Linz, Austria and Altenberger Strasse 96, 4040 Linz, Austria; gerhard.grossboetzl@kepleruniklinikum.at (G.G.); stella.stevoska@kepleruniklinikum.at (S.S.); christian.stadler@kepleruniklinikum.at (C.S.); maximilian.ziernhoeld@kepleruniklinikum.at (M.Z.); petar.noack@kepleruniklinikum.at (P.N.); tobias.gotterbarm@kepleruniklinikum.at (T.G.); Matthias.luger@kepleruniklinikum.at (M.L.)

**Keywords:** osteochondroma, synostosis, bilateral, osteocartilaginous exostosis

## Abstract

*Background and Objectives*: Up until now, only one case of unilateral proximal tibiofibular synostosis caused by osteochondroma has been reported. This report is the first well-documented bilateral case of proximal tibiofibular synostosis caused by an osteochondroma. *Case Report*: A 21-year-old, highly active male patient with bilateral proximal tibiofibular synostosis caused by an osteochondroma suffering from persistent knee pain is presented. As conservative methods had failed, the patient was treated by bilateral open resection of the connecting bone. Histopathological findings confirmed the preoperative diagnosis. The patient returned to sports three weeks after surgery and continued soccer training six weeks after surgery. *Discussion*: The case report presents the successful treatment of a bilateral proximal tibiofibular synostosis caused by an osteochondroma by bilateral open resection of the connecting bone.

## 1. Introduction

Osteochondroma, also called cartilaginous exostosis or osteocartilaginous exostosis, is the most common benign bone tumor. It represents around 30% of all benign and 15% of all osseous neoplasms [1,2]. According to the WHO classification and depending on their radiological appearance, sessile and pediculated types are described. A trilaminate structure (cartilage, cortical, and trabecular bone) is typical [3].

In most cases, the osteochondroma is a solitary, non-hereditary, osseous tumor located in the metaphysis of long bones. The bones that are the most affected are the proximal parts of the tibia, fibula, and humerus as well as the distal femur. The cartilaginous exostosis may also affect the trunk, but this mostly occurs in patients with multiple osteochondromas, which constitutes around 15% of all cases. Tumors develop in childhood, may grow during adolescence, and typically do not enlarge during adulthood.

A transformation from benign osteochondroma to malign chondrosarcoma is reported in about 1% of tumors. The additive risk is summed up in patients with multiple osteochondromas [2,4].

The cartilaginous exostosis is mostly detected by the patient. The most common sign is local swelling. Symptoms depend on their location and may be pain, limitation of joint movement, neurological deficits, and vascular compression.

Symptomless, non-growing osteochondromas do not require surgical therapy due to their benign character.

Excision is only needed if the tumor causes plausible symptoms or suspicious enlargement. A cartilage cap that is more than 2cm thick indicates a malignant transformation [3].

## 2. Materials and Methods

A 21-year-old male patient with persistent knee pain, who was admitted to an outpatient clinic for orthopedic surgery at a university hospital in central Europe, is presented in a case report. The diagnostic pathway, surgical treatment, and outcome are described in detail, including preoperative clinical appearance, plain radiograph, computer tomography (CT), magnetic resonance imaging (MRI), intraoperative images, postoperative plain radiograph, and clinical outcome.

### 2.1. History

The patient described recurrent pain in both of his knees connected to sport activity. He stated that he was very active, with a total of 5–7 h of sports per week, mostly soccer and running. No injuries were reported. Painful sensations worsened during exercise and declined after cessation. Abstention from physical activity led to painlessness, but return to sports activity immediately caused discomfort. The patient was able to locate the painful sensations to the most lateral part of the knee joint, just to the tibiofibular area. He remembered having these problems for at least 12 months.

On his family doctor’s advice, the patient had already undergone physical therapy, medical taping, ultrasound application, and nonsteroidal anti-inflammatory drugs. He stated the that reduction of physical activity was not an option, despite the painless situation during periods of rest. The family doctor indicated plain radiographs of both knees, which showed a synostosis of the proximal tibiofibular joint, most likely caused by an osteochondroma. The subsequent CT and MRI confirmed the diagnosis from the plain radiograph. Due to the long-lasting discomfort during exercise and the diagnosis from the previous imaging, the patient was admitted to the outpatient clinic for orthopedic surgery at a central European university hospital.

The patient stated that he had a condition following congenital valvular stenosis of the pulmonary artery and interventional dilation of the valve at the age of four. Regular cardiological checks were within normal limits.

### 2.2. Presentation of the Patient

The patient was a 21-year-old Caucasian male. Height was 182 cm, weight 82 kg, and body mass index was normal (24 kg/m^2^). Physical examination of both of his knees showed no swelling, no redness, no hyperthermia; no effusion; a range of motion in extension/flexion 0/0/140 degrees; negative meniscal stress tests; stability of cruciate and collateral ligaments; and pressure pain of the proximal fibula. The patient was able to reproduce the painful sensations in weight-bearing, full flexion of the knee (squat position).

### 2.3. Radiological Findings

**Figure 1:**
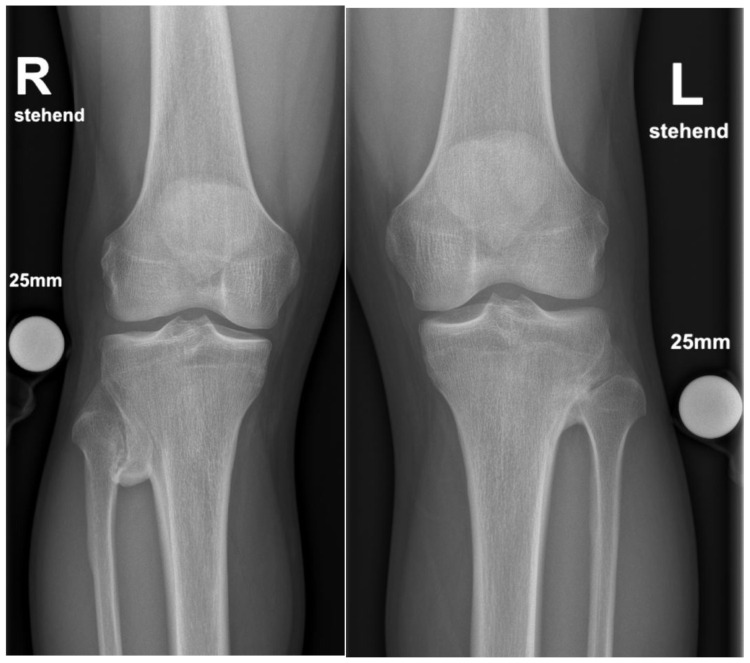
Plain radiograph in a.-p. view showing the bilateral synostosis.

Plain radiographs, performed in anterior-posterior and lateral views of both knees showed a bony excrescence originating from the lateral tibial condyle as well as from the head of the fibula (Figure 1). The joint space was hardly visible. There were no signs of fracture or osteolysis.

**Figure 2:**
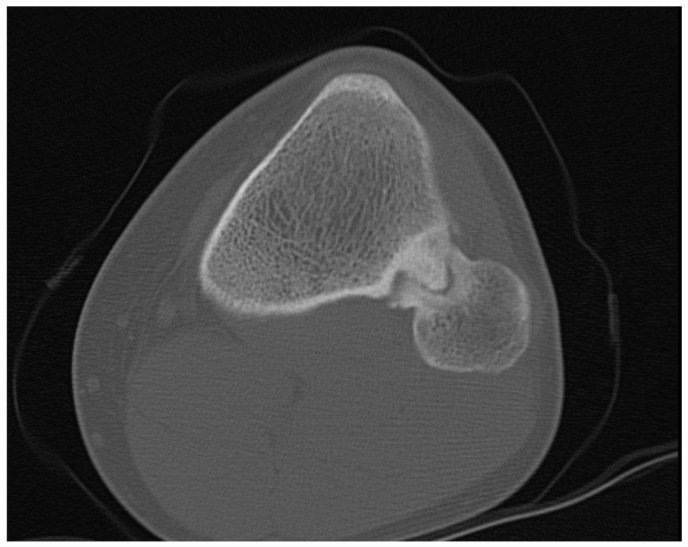
CT scan in horizontal view showing the synostosis of the left side.

The CT scan without contrast of the knees showed partial sclerosis of the protuberance, and the continuity with the medullary cavity was present (Figure 2).

**Figure 3:**
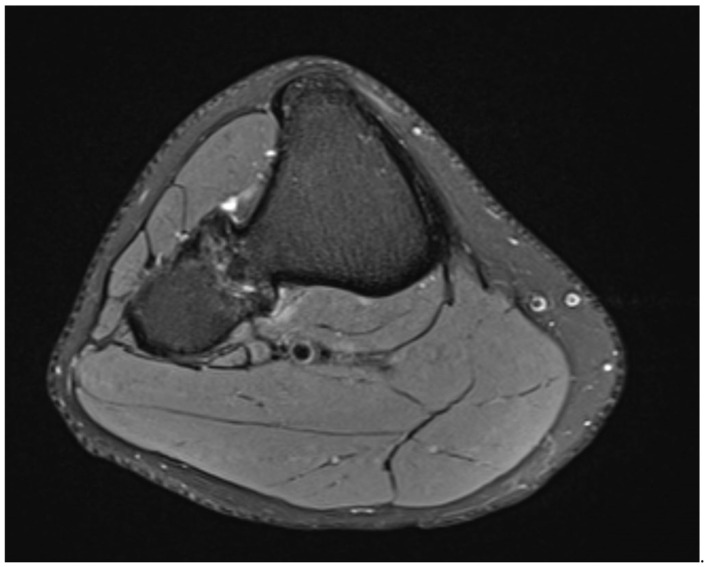
MRI scan in horizontal view showing the synostosis of the right side.

The MRI scan without contrast showed no thickened cartilaginous cap (Figure 3).

The lesions were suspicious for bilateral osteochondroma of the proximal tibiofibular joint and showed no signs of malignancy.

## 3. Results

### 3.1. Procedure

Medical history, physical examination, and results from the medical imaging were discussed with the patient. Due to his long-lasting complaints and the failed conservative therapy, the patient requested surgical therapy. The patient was thoroughly informed about the potential adverse events connected to surgical interventions around the proximal fibula: injury of blood vessels, hematoma, compartment syndrome; injury of the peroneal nerve with palsy and numbness; wound healing disorder, complex regional pain syndrome; osseous reunion of the proximal tibiofibular joint; and symptomatic osteoarthritis of the proximal tibiofibular joint.

Being aware of the mentioned risks, the patient agreed to undergo surgery to resect the connecting bone of the proximal tibiofibular joint.

The anesthesiologist saw the patient, and no contraindication for surgery was indicated.

### 3.2. Surgical Procedure

**Figure 4:**
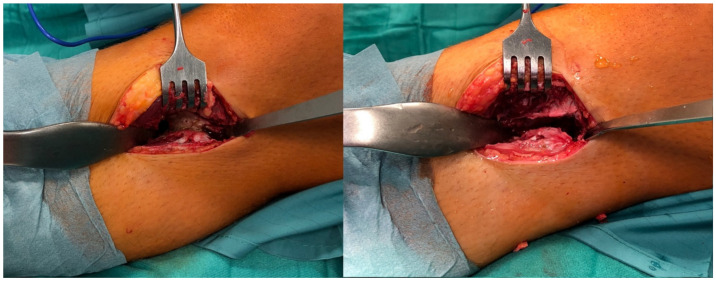
Intraoperative situs before (**left**) and after resection (**right**) of the synostosis of the left side; top = anterior, bottom = posterior, right = proximal, left = distal.

**Figure 5:**
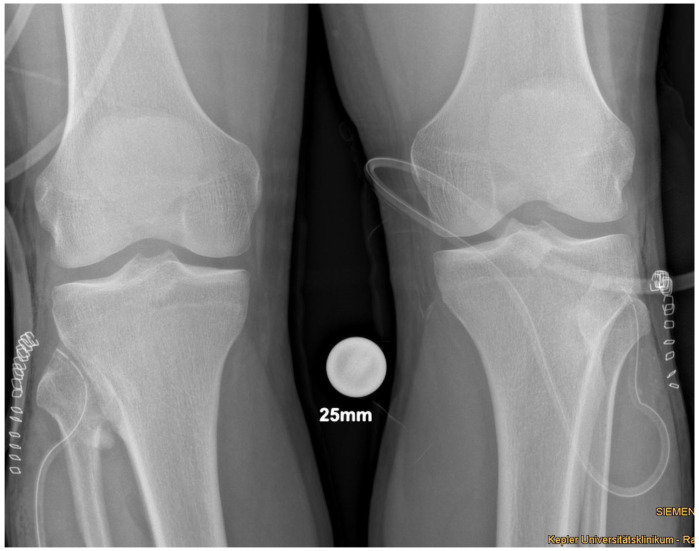
Plain radiograph in a.-p. view after resection.

In supine position and under general anesthesia, both lower extremities were washed and draped in sterile cloth. Surgery was started using Esmarch’s-Method on the left side. The approach was direct anterolateral towards the tibiofibular joint, about 4cm in length. Passing the subcutaneous fat tissue, the crural fascia was dissected, and the proximal part of the anterior tibial muscle was pushed medially and retracted with hooks. Then, the synostosis was visible. The surface of the synostosis was primed with a rasp, and a Hohmann hook was placed proximal and distal. Then, the synostosis was excised with a chisel and a Luer pincer. The gap between the tibia and fibula was considered to be broad enough when a distance of 5mm was reached (Figure 4). Then, the situs was flushed with saline, bone wax was stuck to the open fibular and tibial bone to prevent reossification, and stepwise closure of the wound was performed. A drain was used. The procedure was then performed on the right side in the same way. A postoperative biplane X-ray was performed (Figure 5).

### 3.3. Aftertreatment

The patient was allowed to carefully mobilize with full weight-bearing. The drain was removed on the first postoperative day. The patient was dismissed on the second postoperative day, as checks did now show any signs of compartment syndrome or neurological deficits. The patient needed nonsteroidal anti-inflammatory drugs (Dexibuprofen 400 mg^3^/day) for one week. Sutures were removed by his family doctor two weeks after surgery.

### 3.4. Histological Outcome

Histological workup of the resected material showed an osseocartilaginous lesion consisting of mature and transition bone covered by a cartilage cap with endochondral ossification, the superficial chondrocytes were clustered, and those closer to the transition bone appearing larger, thus mimicking disorganized growth plate cartilage. Focally, a thin layer of perichondrium was detectable. Nuclear atypia and mitoses were absent, in accordance with the diagnosis of an osteochondroma.

### 3.5. Clinical Outcome

The patient started with slow runs 3 weeks after surgery and continued soccer training 6 weeks after surgery. The initial complaints have improved significantly on both knees 6 months after surgery, but slight pain still occurs occasionally after an intense workout.

## 4. Discussion

This is the first well-documented case of a proximal tibiofibular synostosis caused by an osteochondroma.

As this is rarely a symptomatic condition, some of the cases are clearly connected to posttraumatic or iatrogenic states [5,6,7,8]. Conservatively treated tibial fractures [6], surgically treated tibial fractures [5,8], and high tibial osteotomies [8] are reported to be potential causes for proximal tibiofibular synostosis.

Most of the documented cases are thought to be idiopathic, as there are no clear causal events in the patients’ medical histories [5,9,10]. Repetitive microtrauma is thought to be a possible mechanism for proximal tibiofibular synostosis in patients who do not report adequate trauma, as cases of a volleyballer, long-distance runner, and judoka are documented [11,12,13]. Posttraumatic conditions may cause heterotopic ossification, which can be discussed as an underlying mechanism for tibiofibular synostosis [14].

Apart from clear posttraumatic conditions and idiopathic cases, genetic conditions such as the multiple hereditary exostoses syndrome and the 49, XXXXY syndrome may cause a proximal tibiofibular synostosis [15,16,17].

Only one case of a non-traumatic and non-syndromic proximal tibiofibular synostosis is reported in the literature [18].

According to O’Dwyer and Takai (Table 1), proximal tibiofibular synostosis can be distinguished by four different types [9,12].

According to the classification system of O’Dwyer and Takai, the described case can be categorized as Type 4.

The clinical appearance of patients with proximal tibiofibular synostosis may differ a lot. Bone deformities, deviation of axis, and cosmetic salience are documented signs. The most common symptom reported is a painful knee, especially in flexion of the knee and dorsiflexion of the foot [10,12]. Those sensations are interpretated as micromotions in an incomplete synostosis [11].

Ankle pain caused by an impairment of movement in the proximal tibiofibular joint may be one leading symptom [6,18,19]. Erroneous interpretation as radicular sciatic pain and intermittent palsy in the peroneal region are reported [13,20].

Diagnosis is usually possible with X-rays of the knee in two planes and additional CT or MRI scans. Sectional imaging may be helpful in cases where surgical removal of the synostosis is an option for the patient.

As there are no reported negative consequences in patients with conservative treatment, apart from persistent pain, watchful waiting in combination with common conservative treatment may be an option, especially in patients with few symptoms [5,9,10,12].

If conservative treatment (physical therapy, painkillers, adaption of physical activity) is not successful and if painful sensations are described, then surgical removal may be considered.

Techniques that have been reported to be successful are the excision of the synostosis, the resection of the fibular head, the distal osteotomy of the fibula, and the arthrodesis of the proximal tibiofibular joint [6,11,13,18,20,21,22]. Possible complications such as compartment syndrome, lesion of the peroneal nerve, recurrence of the synostosis, symptomatic osteoarthritis of the proximal tibiofibular joint, and the development of cystic lesions need to be determined.

## 5. Conclusions

The case report presents the successful treatment of a bilateral proximal tibiofibular synostosis caused by an osteochondroma by bilateral open resection of the connecting bone.

## Figures and Tables

**Table 1:** Types of proximal tibiofibular synostosis according to O’Dwyer and Takai.

Type 1	Long-distance synostosis in the metaphyseal region without severe deformation
Type 2	Synostosis in the epiphyseal region with proximal bending of the fibula
Type 3	Synostosis in the metaphyseal region with shortening and bending of the fibula
Type 4	Synostosis in the epiphyseal region without severe deformation

## Data Availability

The datasets used and/or analyzed during the current study are available from the corresponding author upon reasonable request. Only the authors have full access to the dataset.

## Abbreviations

a.-p.	anterior-posterior
CT	computer tomography
MRI	magnetic resonance imaging
cm	centimeter
kg	kilogram
m^2^	square meter
